# Longitudinal ventricular cerebrospinal fluid profile in patients with spontaneous subarachnoid hemorrhage

**DOI:** 10.3389/fneur.2022.861625

**Published:** 2022-07-26

**Authors:** Anne Zinganell, Gabriel Bsteh, Franziska Di Pauli, Verena Rass, Raimund Helbok, Janette Walde, Florian Deisenhammer, Harald Hegen

**Affiliations:** ^1^Department of Neurology, Medical University of Innsbruck, Innsbruck, Austria; ^2^Department of Neurology, Medical University of Vienna, Vienna, Austria; ^3^Department of Statistics, Faculty of Economics and Statistics, University of Innsbruck, Innsbruck, Austria

**Keywords:** subarachnoid hemorrhage, cerebrospinal fluid, ventricular, red blood cell, white blood cell, total protein, cytology, longitudinal

## Abstract

**Background:**

Spontaneous subarachnoid hemorrhage (SAH) is a severe neurological disease that frequently requires placement of external ventricular drainage (EVD). Cerebrospinal fluid (CSF) obtained *via* the drain is used to detect potential complications of SAH.

**Objective:**

This study aimed to describe the longitudinal profile of routine CSF parameters in patients with SAH and to identify associations with neurological complications.

**Methods:**

A total of thirty-three patients with spontaneous SAH who required an EVD and had at least three consecutive CSF samples collected over a period of more than 7 days were included in this study.

**Results:**

A median of 6 longitudinally collected CSF samples per patient were available within 1–22 days after SAH onset. Overall, red blood cells (RBC) steadily decreased over time, whereas white blood cells (WBC) and total protein (TP) increased until days 6 and 13, respectively, and decreased thereafter. The estimated decay rates of RBC, WBC, and TP were 28, 22, and 6% per day. Distinct CSF patterns over time were linked to known complications after SAH. Patients with rebleeding showed increased RBC, TP, and phagocytosing cells compared to patients without re-bleeding. For ventriculitis, an elevated cell index with a higher proportion of granulocytes was characteristic. CSF of patients with delayed cerebral ischemia showed increased RBC and WBC compared to patients without DCI. Early CSF WBC and cell index were predictive for the occurrence of DCI and ventriculitis later during the disease course. The amount of daily CSF drainage *via* EVD had no impact on routine CSF parameters.

**Conclusion:**

Longitudinal CSF characteristics are associated with SAH-related complications.

## Introduction

Subarachnoid hemorrhage (SAH) is a severe life-threatening neurological disease resulting from aneurysm rupture in the majority of patients. Due to the blocking of normal cerebrospinal fluid (CSF) circulation resulting from the extravasated blood, a significant proportion of patients develop hydrocephalus and require external ventricular drainage (EVD) (1). Ventricular CSF is used to detect ventriculitis, which occurs in up 10% of patients (2–4). In addition, the CSF profile may also provide insights into mechanisms of SAH-related complications, such as delayed cerebral ischemia (DCI) occurring in ~30% of patients (5). Various biomarkers including endothelins, cytokines, cellular adhesion molecules, or neuronal and glial proteins have been investigated in the CSF of patients with SAH (6), however, studies on the longitudinal changes of routine CSF parameters are still scarce. We hypothesize that CSF parameters follow a different longitudinal evolution in patients with different disease courses. Furthermore, none of these studies considered the impact of CSF loss *via* the EVD, as one might hypothesize that a faster clearance of CSF per time unit might lead to a different longitudinal profile of CSF parameters, i.e., an earlier normalization of CSF red blood cell (RBC) and white blood cell (WBC) counts and CSF total protein (TP) concentration.

The aim of this study was to describe the longitudinal profile of routinely analyzed CSF parameters in patients with SAH requiring EVD placement and to describe distinct changes in patients with rebleeding, and in those developing ventriculitis and DCI. Furthermore, a literature search was performed to summarize current evidence on longitudinal CSF changes in patients with SAH.

## Methods

### Literature search

A literature search in PubMed using the search terms “cerebrospinal fluid” AND “subarachnoid hemorrhage” AND “white blood cells” or “red blood cells” or “total protein” or “granulocytes” or “lymphocytes” or “macrophages” or “siderophages” limited to 11 January 2022 returned 7, 43, 17, 4, 26, 42, and 10 references. Abstracts that primarily did not deal with the longitudinal evolution of these CSF parameters or their usability to predict clinical disease course were excluded. In addition, articles in reference lists of individual papers were selected if considered appropriate. Only original articles written in English were considered.

### Study design, patients, and samples

A total of 33 patients aged ≥18 years with spontaneous SAH who were treated at the Neurointensive Care Unit of the Medical University of Innsbruck in a period of five consecutive years, required ventricular drain and had at least 3 CSF samples collected on different days over a period of more than 7 days were included in this study. CSF collection was performed in all patients for routine diagnostic purposes *via* the EVD and was usually done at fixed time points (twice per week) (2).

Demographic and clinical data of patients with SAH were obtained from the medical charts, as well as from the prospective Innsbruck SAH database.

The occurrence of ventriculitis was defined according to CDC criteria: (i) detection of pathogens in CSF (by culture), or (ii) fulfillment of at least two clinical criteria (fever ≥38°C or headache; neck stiffness or cranial nerve signs), and at least one CSF criteria (increased CSF white blood cells (WBC), elevated CSF protein and decreased CSF glucose; positive CSF gram stain; detection of pathogens in blood by culture or non-culture based microbiologic testing; positive serological result) (7). The clinical signs of headache or neck stiffness were not applied in patients with sedation and analgesia.

Rebleeding was defined as a new/ extended hemorrhage on a CT scan associated with a sudden clinical deterioration at any time during the ICU stay. In patients with sedation and analgesia sudden clinical deterioration or unexplainable changes in blood pressure and heart rate, pupil sizes, or increases in intracranial pressure readings provoked a CT scanning to exclude/confirm suspected rebleeding.

Delayed cerebral ischemia was defined as clinical deterioration with a new focal neurologic deficit, a decrease of greater than or equal to 2 points on the Glasgow Coma Scale, or new infarction on CT or MRI scan not attributable to other causes (8).

### CSF analysis

Determination of all CSF parameters was performed at the Neuroimmunology Laboratory of the Department of Neurology, Medical University of Innsbruck, within 2 h after CSF withdrawal (9). CSF WBC and RBC were counted in a Fuchs-Rosenthal chamber, which has a volume of 3.2 μl. Therefore, counts are reported as “/3” cells (approximate correction for a standard volume of 1 μl) (10). Furthermore, we determined the cell index as previously reported (11):


$$\begin{array}{l} Cell\;index=\;\frac{CSF\;WBC\;\times \;Blood\;RBC\;}{Blood\;WBC\;\times \;CSF\;RBC\;} \end{array}$$

Cerebrospinal fluid total protein concentration was measured by spectrophotometry after incubation of centrifuged CSF with 3% trichloroacetic acid (12).

The cytological preparation followed the recommendations of Lehmitz et al. (13) with slight adaptions. We used 200 μl of undiluted CSF and centrifuged it without the preliminary step of concentration onto the microscope slides. After air-drying, the slides were stained with May-Grünwald-Giemsa staining (14).

**Figure 1:**
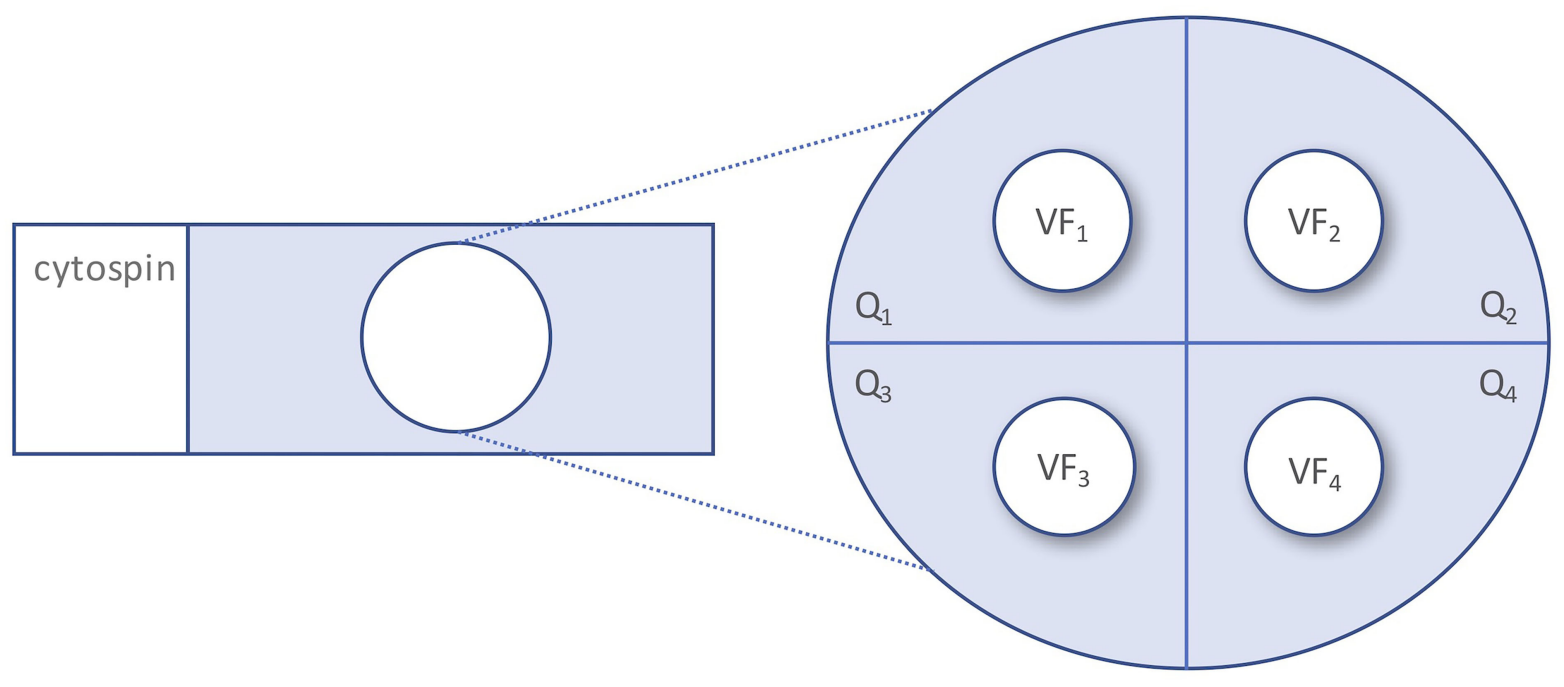
Quantitation of CSF white blood cell subpopulations. CSF, cerebrospinal fluid; Q, quadrant; VF, visual field.

To assess the relative distribution of the WBC subpopulation, we counted the number of granulocytes, lymphocytes, monocytes/macrophages, and erythrophages/siderophages in a single spot of each quadrant of the glass slide at × 400 magnification, calculated the mean number of each cell type and finally the relative proportion (in percentage) for each cell type (Figure 1).

### Statistical analyses

Data are displayed as mean ± standard deviation (SD) or as median and range or interquartile range as appropriate.

To model the decay of RBC, WBC, and CSF total protein over time, we assumed the following function


$$\begin{array}{l} y_{t}=y_{0}e^{-\lambda \cdot t}e^{-\delta \cdot {CSF\text{\_}quantity}_{t}} \end{array}$$

where *y*_*t*_ denotes RBC, WBC, or CSF total protein, respectively at time *t*, *y*_0_ is the starting level of the quantity *y*, λ is the decay parameter due to time (days) and δ is the decay parameter due to cumulative loss of CSF quantity at time *t* (*CSF*−*quantity*_*t*_) *via* the external ventricular drainage (ml).

We estimated the above equation after a logarithmic transformation and used a starting level of *y* for each patient *i* to consider individual heterogeneity (*c*_*i*_):


$$\begin{array}{l} \ln\left(y_{it}\right)=c_{i}-\lambda \cdot t-\delta \cdot {CSF\text{\_}quantity}_{it}+\varepsilon_{it} \end{array}$$

where ε_*it*_ is the remainder noise with the usual ordinary least squares assumptions.

Logistic regressions were computed to identify influential variables for different clinical outcomes (e.g., the overall impact of WBC count on ventriculitis). Repeated measurements were considered by cluster robust standard errors and model assumptions were checked.

Regression analyses with the transformation of the dependent variable if needed (e.g., log transformation of WBC count) and fixed effects for time point and the group at each time point (e.g., patients with and without ventriculitis at time point 1) were used to assess differences of CSF parameters at certain time points between patient groups. Longitudinal measurements were grouped to time points 1–6 according to the following periods: days 1–4, 5–8, 9–11, 12–15, 16–18, and 19–22.

Receiver operating characteristic analyses were performed to assess the value of CSF parameters at time point 1 to predict the occurrence of SAH complications later during follow-up. Sensitivities, specificities, and area under the curve (AUC) were provided.

The significance level was 5%. All computations were done with R Core Team (15) and the package survival (16, 17).

### Ethics

The conduct of the study was approved by the local ethics committee of the Medical University of Innsbruck (approval number AM4091-292/4.6). Written informed consent was obtained according to Austrian law and in accordance with the Declaration of Helsinki.

## Results

### Literature search

Few studies on the longitudinal evolution of routine parameters in ventricular CSF (i.e., RBC count, WBC count including its subpopulations, and total protein) were identified. The main findings were that RBC and CSF TP decrease with SAH disease duration (18–20), that WBC and CSF TP are higher in patients with ventriculitis (21), that CSF neutrophils are associated with the occurrence of cerebral vasospasm (22) and that CSF TP is higher in patients with DCI (20). Further details on the results of these studies are summarized in Supplementary Table S1.

### Longitudinal evolution of CSF parameters

A total of 33 patients with a mean age of 57 ± 14 years comprising 70% of women had 177 CSF samples collected *via* the EVD within 1–22 days after SAH onset. A median of 6 (range 3–6) longitudinally collected CSF samples were available per patient. For further detailed clinical characteristics, we refer to Table 1.

**Table 1:** Demographic and clinical characteristics.

Age (years) at SAH onset, mean ± SD	57 ± 14
Sex (female), *n* (%)	23 (70)
**Hunt & Hess at admission**, ***n*** **(%)**	
Grad I-II	3 (9)
Grad III	6 (18)
Grad IV-V	24 (73)
**External ventricular drainage**, ***n*** **(%)**	33 (100)
CSF volume drained per day (ml), median (IQR)	199 (159–241)
**Time from SAH onset to CSF** **sampling (days), median (range)**^1^	
Time point 1	3 (1-4)
Time point 2	6 (5-8)
Time point 3	10 (9-11)
Time point 4	13 (12-15)
Time point 5	17 (16-18)
Time point 6	20.5 (19-22)
**Neurological complications**	
**Re-bleeding**, ***n*** **(%)**	6 (18)
Time of occurrence (days after SAH onset), median (IQR)	2 (0.5–16.5)
**Ventriculitis**, ***n*** **(%)**	5 (15)
Time of occurrence (days after EVD insertion), median (IQR)	13 (6.5–25.5)
Evidence of CSF pathogen, *n* (%)^2^	5 (100)
Antibiotic treatment before diagnosis of ventriculitis, *n* (%)	3 (60)
**Delayed cerebral ischemia**, ***n*** **(%)**	12 (36)
Time of occurrence (days after SAH onset), median (IQR)	8 (2.5–12.5)
**Treatment**	
Coiling, *n* (%)	19 (57.6)
Time to coiling (days after SAH onset), median (IQR)	0 (0–0)
Clipping, *n* (%)	11 (33.3)
Time to clipping (days after SAH onset), median (IQR)	0 (0–0)
No aneurysma detection, *n* (%)	3 (9.1)
Time from coiling/clipping to first CSF sampling (days), median (IQR)	3 (1-3)

^1^* At time points 1–6, a total of 28, 33, 30, 31, 27, and 28 CSF samples were available*.

*^2^ Staphylococcus epidermidis (n = 2), coagulase-negative staphylococcus (n = 3)*.

*CSF, cerebrospinal fluid; EVD, external ventricular drainage; IQR, interquartile range; n, number; SAH, subarachnoid hemorrhage; SD, standard deviation*.

**Figure 2:**
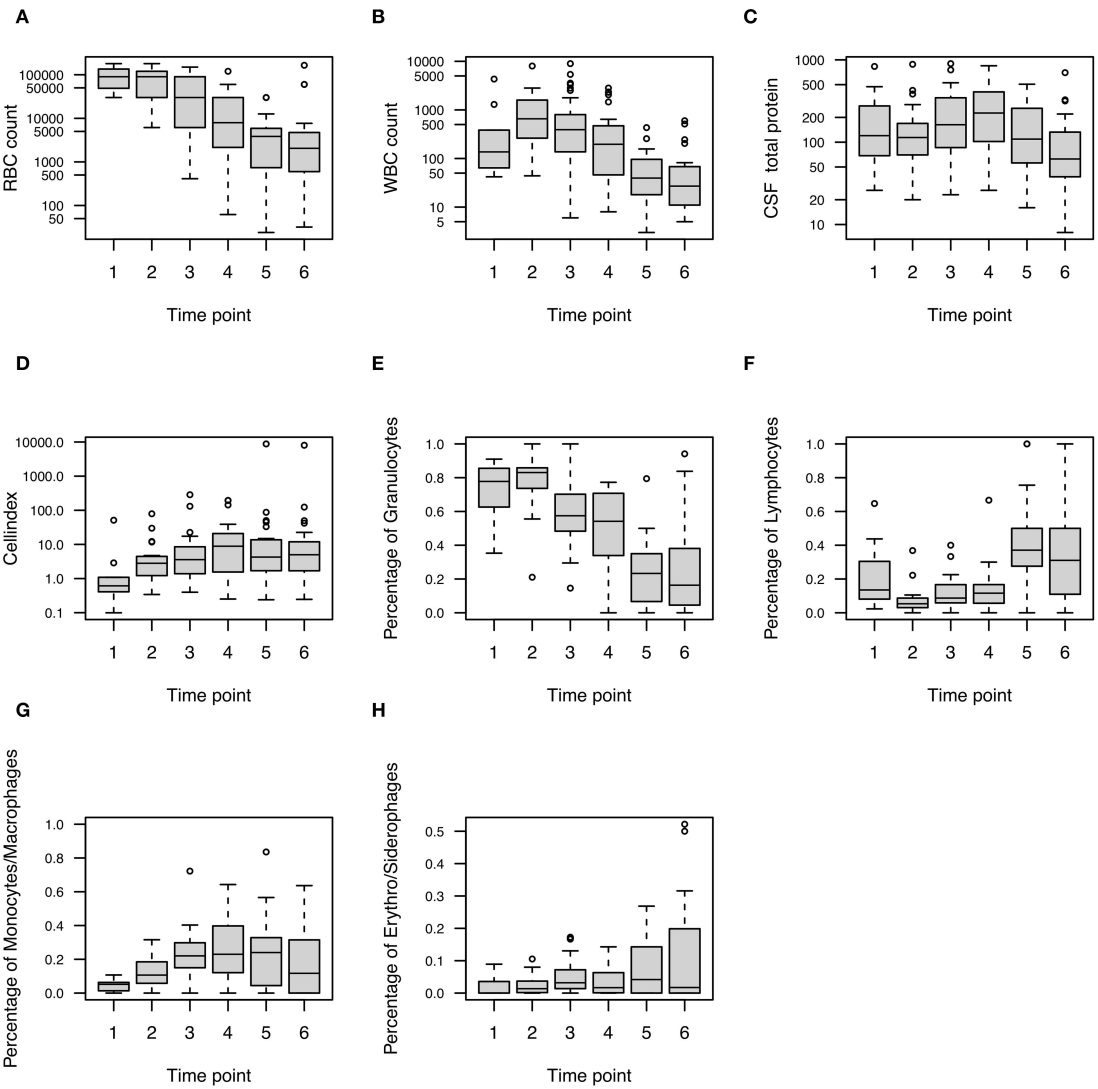
Longitudinal profile of CSF parameters in patients with SAH. Boxplots show **(A)** RBC, **(B)** WBC, **(C)** CSF total protein, **(D)** cell index, **(E)** granulocytes, **(F)** lymphocytes, **(G)** monocytes/ macrophages, and **(H)** erythrophages/ siderophages at six-time points after the onset of SAH (i.e., after median 3, 6, 10, 13, 17, and 20.5 days). CSF, cerebrospinal fluid; RBC, red blood cells; SAH, subarachnoid hemorrhage; WBC, white blood cells.

**Figure 3:**
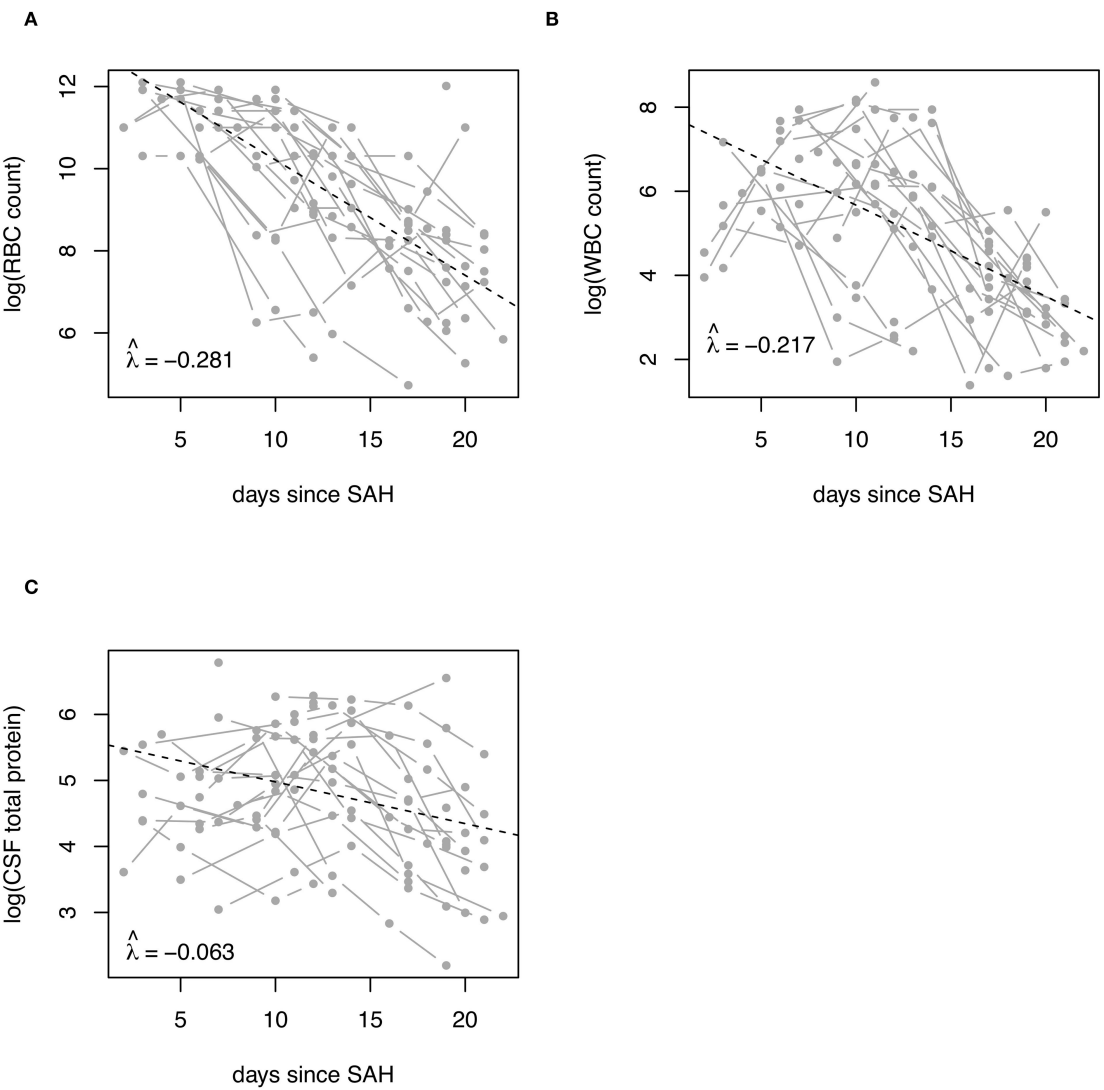
Decay rates of CSF RBC, WBC, and total protein in patients with SAH. Decay rates of **(A)** RBC, **(B)** WBC, and **(C)** total protein in ventricular CSF of patients with SAH are shown. Decay rates were adjusted for the amount of CSF discarded *via* the ventricular drain. CSF, cerebrospinal fluid; RBC, red blood cell count; SAH, subarachnoid hemorrhage; WBC, white blood cell count.

Overall, CSF RBC count steadily decreased during the disease course, whereas WBC count and CSF total protein first showed an increase reaching the highest values after a median of 6 and 13 days, respectively, before the eventual drop (Figures 2A–C). CSF RBC, CSF WBC, and CSF total protein decreased by 28.1, 21.7, and 6.3% per day, respectively (Figures 3A–C). The time decay parameter was statistically significant (*P* < 0.001), while no evidence for a statistically significant decay parameter due to the cumulative loss of CSF quantity (*via* the EVD) was found.

The cell index increased until day 13 and declined afterward (Figure 2D). WBC subpopulations showed a differentiated pattern. Granulocytes were highest after a median of 6 days, while lymphocytes, monocytes/macrophages, and erythro-/siderophages peaked later during the disease course after a median of 17 days (Figures 2E–H).

### Association of CSF parameters with clinical SAH-related complications

Cerebrospinal fluid parameters showed a distinct longitudinal development associated with the clinical disease course.

A total of 6 (18%) patients had re-bleeding, in median 2 days after SAH onset. Two patients had re-bleeding before clipping and one patient had insufficient aneurysm coiling. Two patients with re-bleeding showed still sufficiently occluded aneurysms in control digital subtraction angiography. One patient had neither any detectable aneurysm nor any intervention. Overall, the type of intervention was not associated with re-bleeding (*P* = 0.236). Patients with re-bleeding showed higher RBC counts, CSF total protein concentration (Supplementary Figure S1A), and a higher relative proportion of phagocytosing cells, i.e., monocytes/ macrophages and erythro-/siderophages, during the disease course compared to patients without re-bleeding (Supplementary Figure S1B). While RBC count and CSF total protein were higher already at the time of the first CSF collection (Supplementary Figure S1A), a relevant increase of phagocytosing cells occurred with a delay of several days (Supplementary Figure S1B).

Patients who developed ventriculitis (*n* = 5, 15%; in median 13 days after EVD insertion) had elevated percentages of granulocytes throughout the whole observation period, but lower percentages of phagocytosing cells compared to patients without ventriculitis (Supplementary Figure S2B). Whereas WBC counts did not show a clear pattern, the cell index was consistently elevated at every time point in patients with ventriculitis (Supplementary Figure S2A). A cell index >2 at the time of first CSF collection showed a sensitivity of 25% and specificity of 93.3% to predict ventriculitis (*P* = 0.107; AUC = 0.6). CSF total protein showed higher values from day 10 on (Supplementary Figure S2A). The type of aneurysm occlusion showed no difference with regard to developing ventriculitis (Coiling: 11%, Clipping 9%, *P* = 0.9).

Cerebrospinal fluid of patients who developed DCI (in median 8 days after SAH onset) had higher RBC counts and contained more WBC compared to those without DCI (Supplementary Figure S3A). WBC count was significantly elevated already at the time of first CSF collection, and a WBC count >1,000 showed a sensitivity of 62.5% and specificity of 80% to predict DCI (AUC = .767, *P* = 0.039). The WBC subpopulations did not differ between patients with and without DCI (Supplementary Figure S3B). There was no statistically significant difference in the type of intervention for developing DCI (Coiling: 42%, Clipping 36%, *P* = 0.757).

## Discussion

Cerebrospinal fluid analysis is used in patients with SAH and EVD to detect drain-associated ventriculitis (3, 4). Besides that, CSF provides an opportunity for early identification of patients who develop other complications of SAH, such as DCI (6).

Previous studies reported the main CSF findings in patients with SAH and its complications. However, many of these studies are limited by the application of cross-sectional design, varying patient inclusion criteria, and revealed to some extent inconsistent findings. As different complications typically occur at different time points during the SAH disease course, e.g., rebleeding in the first days (23), DCI within 3–10 days (5), and ventriculitis mostly 6–10 days after EVD insertion (24–26), the time of CSF sampling is critical when interpreting CSF results. Studies with a longitudinal design are scarce and often focused only on certain CSF parameters (e.g., only CSF protein) and/ or certain clinical complications (e.g., only ventriculitis) (Supplementary Table S1). Furthermore, none of these studies considered the impact of CSF loss *via* the EVD. To overcome these methodical limitations, we applied a longitudinal study design, obtained the whole panel of routine CSF parameters, investigated the associations with SAH complications, and also considered the possible impact of CSF loss *via* the EVD.

Here, we showed that RBC, WBC, and CSF total protein followed a different temporal evolution. Whereas CSF RBC steadily decreased during the disease course, WBC count and CSF total protein first showed an increase reaching the highest values in the median after 6 and 13 days, respectively, before the eventual drop. These findings largely confirm the findings of previous studies (18, 20). For the first time, we also estimated decay rates for RBC, WBC, and CSF total protein, i.e., the relative percentage decrease of these parameters per day. Decay rates of RBC and WBC were similar (−0.281 and −0.217), while that of CSF TP was significantly lower (−0.063). This probably reflects different pathophysiological processes, where cells underlie similar degradation, while CSF TP indicates blood-CSF-barrier dysfunction. Physiological CSF drainage either slowly normalizes during the SAH disease course or even not to that extent in a subgroup of patients, who develop chronic hydrocephalus requiring ventriculoperitoneal shunting.

Decay rates might be useful for further clinically relevant research, e.g., for the discrimination of patients with (CT negative) SAH and traumatic lumbar puncture. In patients with SAH and available lumbar CSF, the RBC count at the time of the bleeding might be estimated from the RBC count determined at the time of lumbar puncture, using the decay rate and the respective time from symptom onset, i.e., adjusting for the *in vivo* cell degradation over time. This is relevant, because a significant proportion of patients with thunderclap headaches—at risk of having suffered an intrathecal hemorrhage—present themselves with delay, sometimes several days, and up to 20% of patients experience a “warning leak” before the eventual major SAH onset (27). This approach would possibly allow the determination of RBC cut-off levels adjusted for time since the event that better distinguish between traumatic tap and (CT negative) SAH. Currently available studies comparing RBC counts of traumatic lumbar puncture with patients with SAH included patients who had bleeding onset already days before sampling (28) and thus probably underestimate RBC counts.

Furthermore, we addressed the impact of CSF loss *via* the EVD, as different CSF clearance might lead to a different longitudinal CSF profile. We did not observe any impact of CSF quantity loss on CSF parameters; however, we have to admit that this might be confounded by the small number of patients.

With regard to the different SAH complications, we observed distinct CSF patterns. In bacterial ventriculitis, previous studies reported elevated WBC (21, 24, 29–39) and an increased proportion of polymorphonuclear cells in CSF (32, 37, 40) compared to SAH without ventriculitis. Also, CSF total protein concentration and cell index, which corrects WBC count for blood contamination (11, 31, 41, 42), were significantly higher in patients with ventriculitis (21, 31, 33, 37, 38, 43). In this study, we largely confirmed these findings, and additionally, we found that cell index was more suitable than WBC count to indicate ventriculitis. While WBC showed a considerable variation over time (even with lower median values in the ventriculitis group at certain time points), cell index was consistently elevated throughout the whole observation period. An elevated cell index (>2) even at the time of first CSF collection (in median 3 days after SAH onset) indicated the development of ventriculitis (occurring in the median of 13 days after EVD insertion) with a specificity of 93.3%. Suitability of cell index might be attributed to the correction for WBC introduced into CSF by the bleeding itself rather than by recruitment through the inflammatory process during the SAH disease course (11). Interestingly, we observed that the proportion of phagocytosing cells was lower in patients with ventriculitis. A possible explanation might be that the relative proportion of certain WBC subpopulations (i.e., monocytes/macrophages and erythro-/siderophages) decreases due to the relative increase of other subpopulations (i.e., granulocytes).

In terms of clinical applicability, we fully agree with previous consensus papers stating that routine CSF measures have limited diagnostic value in differentiating between ventriculitis and sterile inflammation in patients with external CSF drains (4). It is unquestioned that CSF culture is the most important test to establish the diagnosis of drain-associated ventriculitis (4), but culture results take time. Clinical characteristics, such as neck stiffness and altered mental status, may be difficult to assess in many patients because of the primary neurologic disease and sedation and analgesia, respectively. Besides CSF/serum glucose ratio and CSF lactate, CSF WBC count and cell index constitute early diagnostic parameters. We also want to point out that the distribution of WBC subpopulations seems helpful in distinguishing between sterile inflammation and bacterial drain-associated ventriculitis.

Aseptic inflammation that is caused by the degradation of intracranial blood (or as a consequence of a neurosurgical procedure) plays a crucial role in the pathophysiology of SAH and the occurrence of cerebral vasospasm and DCI, respectively (44). WBC are hypothesized to cross the arterial walls and infiltrate the subarachnoid blood clot, secreting cytokines and initiating different processes (5, 6). In line with this, we found higher WBC even in the first collected CSF samples (in median 3 days after SAH onset) in patients who later developed DCI (in median 8 days after SAH onset) compared to those without DCI. With regard to the leukocyte subset, previous studies reported increased granulocyte content in the CSF of patients with vasospasm (22). To the best of our knowledge, studies relating CSF leukocyte subsets with DCI do not exist yet. Here, we could not observe a predominance of granulocytes in the CSF of patients with DCI as compared to patients without DCI. Studies on cytokine profiles in SAH showed an elevation of Tumor necrosis factor-α and Interleukin-6 in patients with cerebral vasospasm or DCI suggesting a role of lymphocytes and monocytes rather the granulocytes (6). Further studies are needed to investigate the immune response after bleeding—as it has recently been done in patients with intracerebral hematoma by transcriptional cell profiling—as well as its impact on the evolution of DCI (45).

There are several limitations of the present work. First, this was a retrospective study of prospectively collected data with all inherent limitations. Second, the requirement of a minimum number of serial samples at the specified time points per patient reduced the number of subjects eligible for inclusion. Consequently, even clear differences were not statistically significant between groups at every time point. Furthermore, our cohort does not reflect the whole spectrum of patients with SAH, as we included only patients requiring EVD introducing a selection bias toward patients with a more severe disease course, and our results may not be transposed to patients without requiring an EVD. Also, CSF sampling was performed for routine diagnostic purposes (usually at fixed time points twice per week), but not on dedicated days with regard to SAH onset. The practice of regular routine CSF sampling might also explain a higher rate of ventriculitis. Due to the small number of patients, multivariate analysis adjusting for between-group differences, such as age, disease severity, the occurrence of more than one complication at the same time, type of intervention (coiling vs. clipping), and differences in CSF parameters, was not feasible.

In conclusion, knowledge about the time window for certain complications and the longitudinal evolution of CSF parameters helps interpret CSF results in patients with EVD. As reliable and validated cut-off values for the main CSF parameters, e.g., WBC count or cell index, are still lacking, it is up to clinicians to include several variables ranging from clinical symptoms to certain CSF patterns in order to judge whether e.g., a bacterial ventriculitis is present or not. Although this study is confirmatory to some extent, there are also new findings worth to be considered in clinical decision-making, like the predominance of granulocytes in patients with ventriculitis. A sound and interesting basis for future research provide the applicability of cellular decay rates.

## Data availability statement

The raw data supporting the conclusions of this article will be made available by the authors, without undue reservation.

## Ethics statement

The conduct of the study was approved by the local Ethics Committee of the Medical University of Innsbruck (approval number AM4091-292/4.6). Written informed consent was obtained according to Austrian Law and in accordance with the Declaration of Helsinki.

## Author contributions

AZ has participated in the acquisition of the data and drafting of the manuscript. GB, FDP, and FD has participated in reviewing the manuscript for intellectual content. VR has participated in the acquisition of the data and reviewing the manuscript for intellectual content. RH has participated in the conception and design of the study and reviewed the manuscript for intellectual content. JW has participated in statistical analysis of the data and reviewed the manuscript for intellectual content. HH has participated in the conception and design of the study, acquisition of the data, statistical analysis of the data, and the drafting of the manuscript. All authors contributed to the article and approved the submitted version.

## Conflict of interest

Author AZ has participated in meetings sponsored by, received speaking honoraria or travel funding from Biogen, Merck, Sanofi-Genzyme, and Teva. GB has participated in meetings sponsored by, received speaker honoraria or travel funding from Biogen, Celgene-BMS, Lilly, Merck, Novartis, Sanofi-Genzyme, and Teva and received honoraria for consulting Biogen, Celgene-BMS, Merck, Novartis, Roche, Sanofi-Genzyme, and Teva. FDP has participated in meetings sponsored by, received honoraria (lectures, advisory boards, consultations) or travel funding from Almirall, Bayer, Biogen, Celgene-BMS, Merck, Novartis, Sanofi-Genzyme, Sandoz, Roche, and Teva. Her institution has received research grants from Roche. FD has participated in meetings sponsored by or received honoraria for acting as an advisor/speaker for Alexion, Almirall, Biogen, Celgene, Genzyme-Sanofi, Merck, Novartis Pharma, Roche, and Teva. His institution has received research grants from Biogen and Genzyme Sanofi. He is section editor of the MSARD Journal (Multiple Sclerosis and Related Disorders). HH has participated in meetings sponsored by, received speaker honoraria or travel funding from Bayer, Biogen, Celgene, Merck, Novartis, Sanofi-Genzyme, Siemens, Teva, and received honoraria for acting as consultant for Biogen, Celgene, Novartis and Teva. The remaining authors declare that the research was conducted in the absence of any commercial or financial relationships that could be construed as a potential conflict of interest.

## Publisher's note

All claims expressed in this article are solely those of the authors and do not necessarily represent those of their affiliated organizations, or those of the publisher, the editors and the reviewers. Any product that may be evaluated in this article, or claim that may be made by its manufacturer, is not guaranteed or endorsed by the publisher.
