# Supplementary material for: Longitudinal ventricular cerebrospinal fluid profile in patients with spontaneous subarachnoid hemorrhage
Source: Front Neurol. 2022 Jul 26;13:861625. doi: 10.3389/fneur.2022.861625 (PMC9360751; doi:10.3389/fneur.2022.861625)
Supplement: Supplementary file 1 [file Data_Sheet_1.PDF]

Figure S1: Longitudinal evolution of CSF parameters stratified by the occurrence of rebleeding

(A)

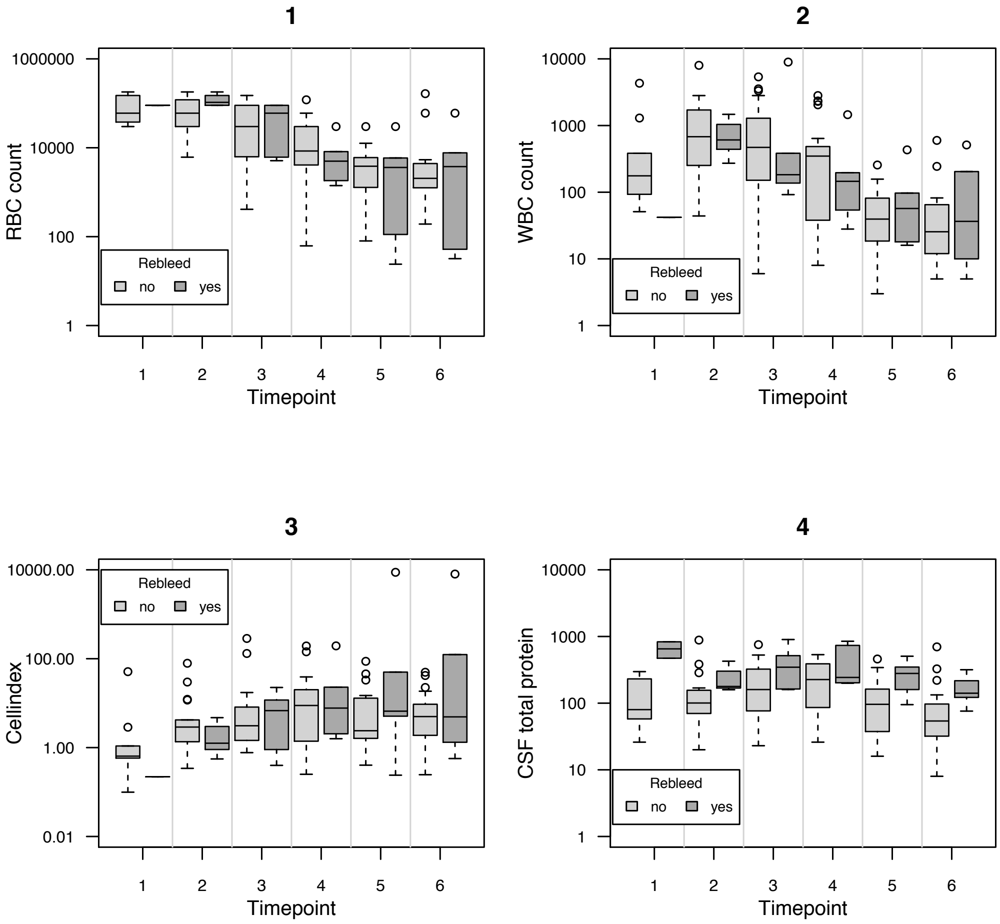

(1) RBC counts were higher in patients with rebleeding at time points 1, 2, 3 and 6 compared to patients without rebleeding (reaching statistical significance at time points 3 and 6 [p<0.001 and p=0.002]).
(2) WBC count was statistically significantly higher in patients with rebleeding at time points 5 and 6 (p=0.002 and p<0.001).
(3) Cell index was similar between patients with and without rebleeding (overall p=0.666).
(4) CSF total protein showed consistently elevated values in patients with rebleeding (reaching statistical significance at time points 1, 5 und 6 [0.009, 0.028, 0.002]).

RBC and WBC counts are reported as “/3”. CSF total protein concentration is given in mg/dl. Cell index was calculated according to Pfausler et al. Acta Neurochir 2004;146: 477-481.

Abbreviations: CSF, cerebrospinal fluid; RBC, red blood cell, WBC, white blood cell

(B)

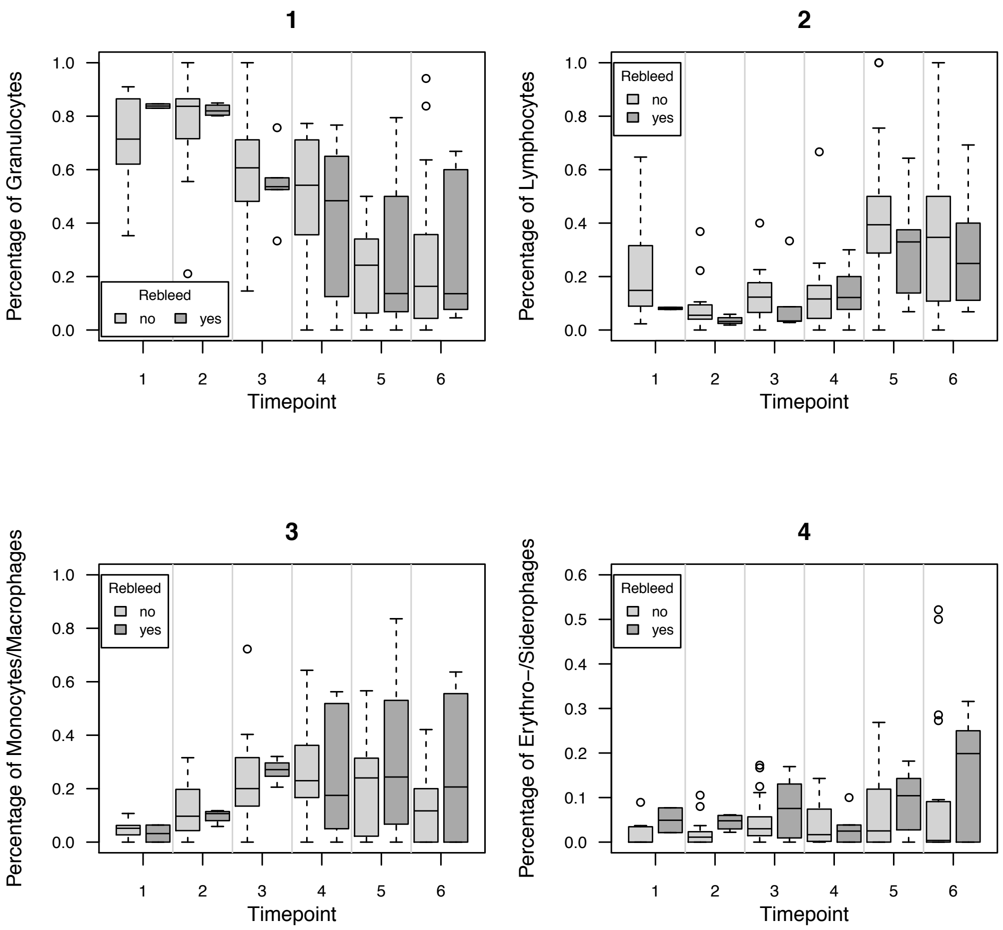

(1) Percentage of granulocytes was lower in patients with rebleeding between time points 2 and 6 (reaching statistical significance at time points 5 and 6 [p=0.021 and p=0.019]).

(2) Percentage of lymphocytes was lower in patients with rebleeding between time points 1 and 6 (reaching statistical significance at time point 5 [p=0.036]).

(3) Percentage of monocytes/ macrophages was higher in patients with rebleeding at time points 2, 3, 5 and 6 (reaching statistical significance at time points 2 and 3 [p=0.017 and p=0.004]).

(4) Percentage of erythrophages/ siderophages was consistently increased in patients with rebleeding throughout the whole observation period (without reaching statistical significance [overall p= 0.156]).

The y-axis represents the percentage of WBC subpopulations (e.g., of granulocytes). This percentage shows the proportion of the specific WBC subpopulation on the total WBC number which both were counted at 400x magnification (for details refer to Methods section).

Figure S2: Longitudinal evolution of CSF parameters stratified by the occurrence of ventriculitis

(A)

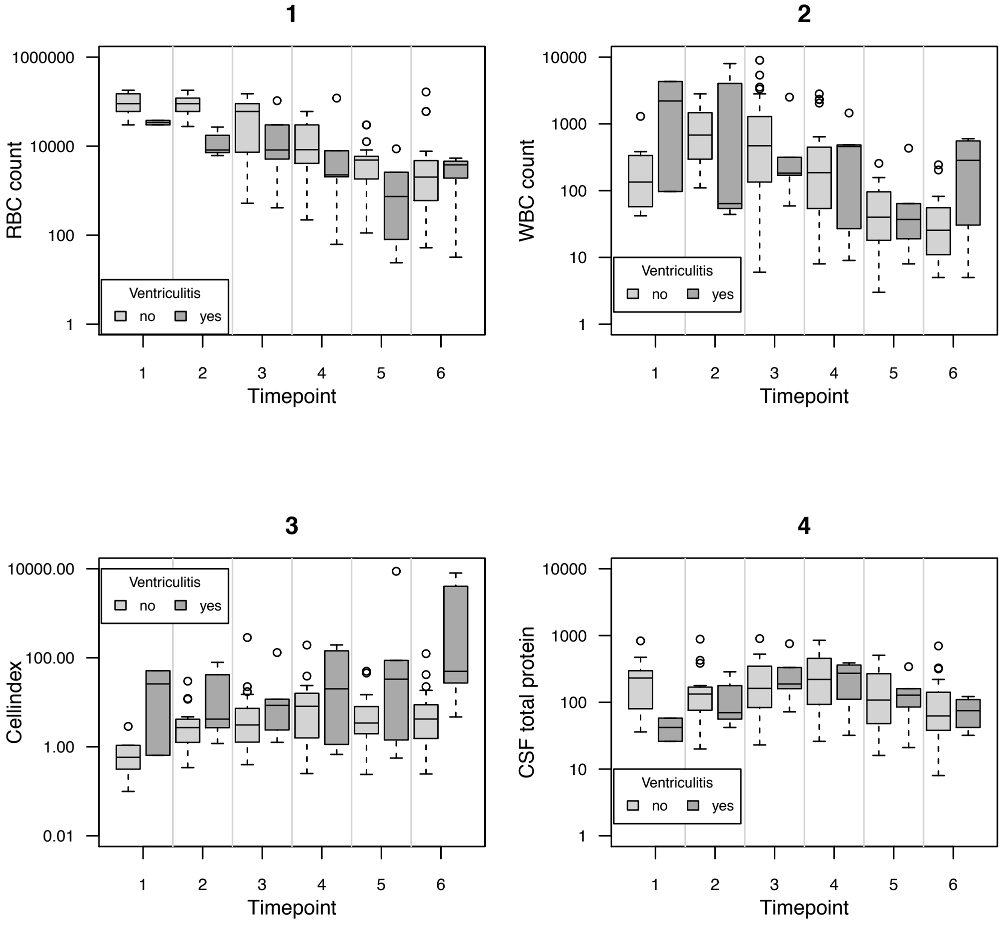

(1) RBC count was similar between patients with and without ventriculitis (overall p=0.340). Only at time point 6, RBC count was statistically significantly elevated in patients with ventriculitis (p=0.013).

(2) WBC count was statistically significantly higher in patients with ventriculitis at time point 6 ($p<0.001$).

(3) Cell index showed was consistently increased in patients with ventriculitis throughout the whole observation period (without reaching statistical significance [overall p=0.243]).

(4) CSF total protein was higher in patients with ventriculitis between time points 3 and 6 (without reaching statistical significance [overall p=0.549]).

RBC and WBC counts are reported as "/3". CSF total protein concentration is given in mg/dl. Cell index was calculated according to Pfausler et al. Acta Neurochir 2004;146: 477-481.

Abbreviations: CSF, cerebrospinal fluid; RBC, red blood cell, WBC, white blood cell

(B)

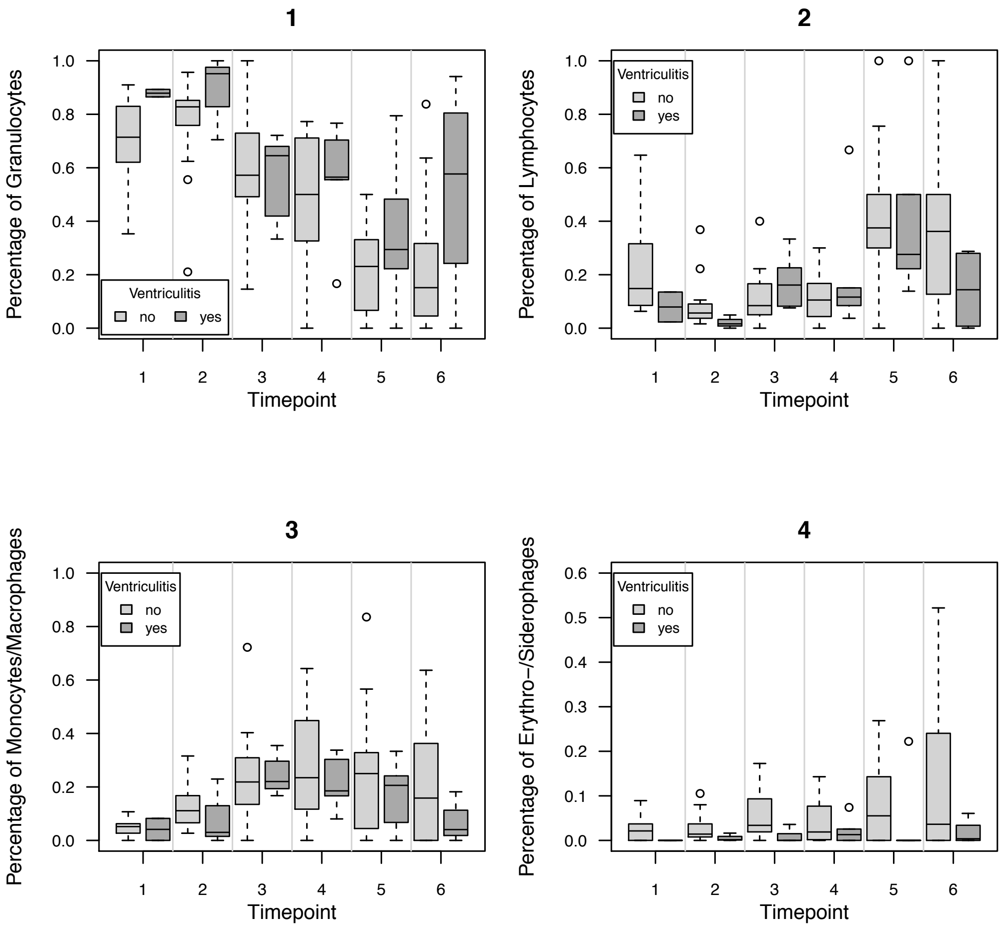

(1) Percentage of granulocytes was consistently increased in patients with ventriculitis throughout the whole observation period (reaching statistical significance at time point 4 [0.023], time point 5 [0.001] and time point 6 [<0.001]).

(2) Percentage of lymphocytes was statistically significantly higher at time points 3 and 4 (p=0.010 and 0.049), but decreased at time point 5 and 6 (p=0.355 and p=0.009).

(3) Percentage of monocytes/ macrophages was statistically significantly lower at time point 4 [p<0.001] and time points 5 and 6 [both p=0.005]).

(4) Percentage of erythrophages/ siderophages was consistently decreased throughout the whole observation period in patients with ventriculitis (reaching statistical significance at time points 3 and 6 [p=0.002 and p<0.001]).

The y-axis represents the percentage of WBC subpopulations (e.g., of granulocytes). This percentage shows the proportion of the specific WBC subpopulation on the total WBC number which both were counted at 400x magnification (for details refer to Methods section).

Figure S3: Longitudinal evolution of CSF parameters stratified by the occurrence of delayed cerebral ischemia

(A)

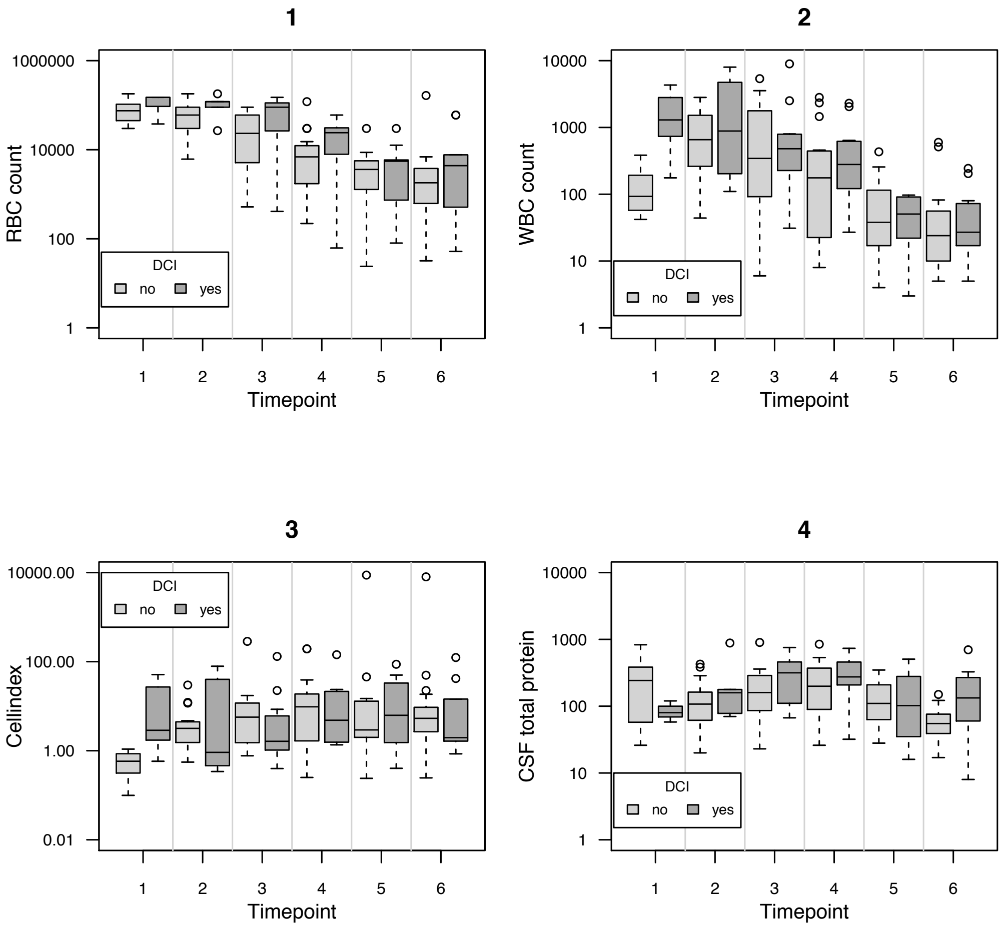

(1) RBC counts were consistently elevated in patients with DCI throughout the whole observation period (reaching statistical significance at time points 2, 3, 4 and 5 [all p<0.001]).

(2) WBC count was consistently elevated in patients with DCI throughout the whole observation period (reaching statistical significance at time point 1 [p=0.021], time point 5 [p=0.008] and time point 6 [p=0.006]).

(3) Cell index was similar between patients with and without DCI (overall p=0.139).

(4) CSF total protein was similar between patients with and without DCI (overall p=0.178)

RBC and WBC counts are reported as “/3”. CSF total protein concentration is given in mg/dl. Cell index was calculated according to Pfausler et al. Acta Neurochir 2004;146: 477-481.

Abbreviations: CSF, cerebrospinal fluid; RBC, red blood cell, WBC, white blood cell

(B)

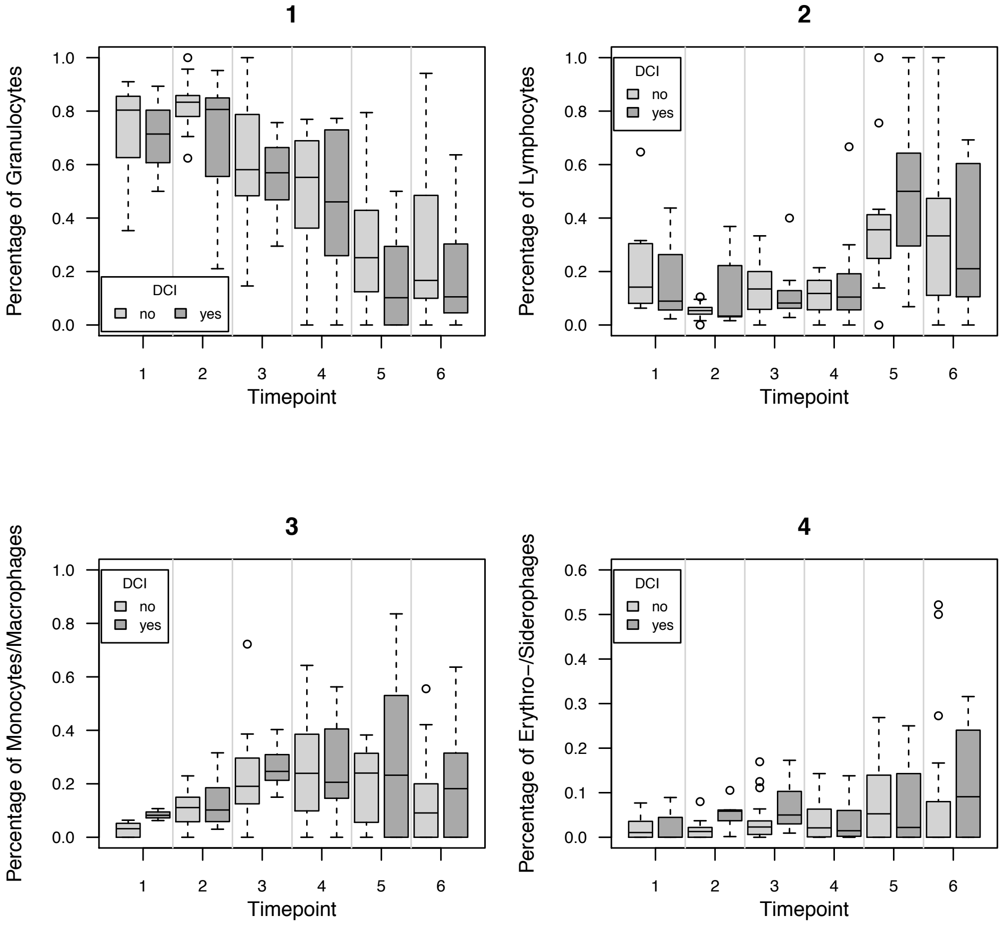

(1) Percentage of granulocytes was similar between patients with and without DCI (overall p=0.074).
(2) Percentage of lymphocytes was similar between patients with and without DCI (overall p=0.412).
(3) Percentage of monocytes/ macrophages was similar between patients with and without DCI (overall p=0.370).
(4) Percentage of erythrophages/ siderophages was similar between patients with and without DCI (overall p=0.468).

The y-axis represents the percentage of WBC subpopulations (e.g., of granulocytes). This percentage shows the proportion of the specific WBC subpopulation on the total WBC number which both were counted at 400x magnification (for details refer to Methods section).
