# Supplementary material for: Longitudinal ventricular cerebrospinal fluid profile in patients with spontaneous subarachnoid hemorrhage
Source: Front Neurol. 2022 Jul 26;13:861625. doi: 10.3389/fneur.2022.861625 (PMC9360751; doi:10.3389/fneur.2022.861625)
Supplement: Supplementary file 2 [file Data_Sheet_2.PDF]

Table S1: Reports on longitudinal ventricular CSF profile

Reference	Study design	No. of patients/ samples	Diagnosis	Age (years)§	Sex (female)#	Sampling interval & duration	CSF parameters	Main findings
**Koopman** et al. 2020 32480074	R, SC	209 / 306	s-SAH (Samples from LP, ELD and EVD)	60 ± 13	140 (67)	Until Day 20	WBC<br>RBC<br>RBC/WBC ratio<br>TP<br>Glucose	CSF TP and RBC/WBC ratio decrease over time<br>WBC peaked at day 6<br>CSF glucose remained normal
**Lenski** et al 2019 30268545	R, SC	63 / NA	s-SAH with EVD (Groups defined by need of VPS during FU)	55 (18-80)	42 (67)	Daily until Day 17	RBC<br>TP	RBC and CSF TP decrease over time<br>RBC↑[1] & CSF TP↑[2] in patients with shunt-dependent hydrocephalus
**Lenski** et al 2018 29494414	R, SC	63 / NA	s-SAH with EVD (Groups defined by ventriculitis and non-ventriculitis)	53 ± 12[3]<br>60 ± 12[4]	42 (67)	Daily	WBC<br>TP	WBC↑ and CSF TP↑ in patients with ventriculitis
**Nadkarni** et al 2020 31932209	P, SC	130 / NA	s-SAH with EVD (Groups defined by outcome[5] or DCI[6])	55 ± 15	90 (69)	Daily until Day 7[7]	TP	CSF TP decreases over time<br>CSF TP↑ in patients with DCI or worse outcome
**Provencio** et al 2010 19967568	S, SC	70 / NA	s-SAH with EVD (Groups defined by VS and non-VS)	57 (32-89)	44 (63)	Daily until Day 3	Neutrophils	CSF neutrophils ↑ in patients with VS<br>(>62% CSF neutrophils predictor for VS)

Legend:

Data are shown as § median (range) or mean (± standard deviation), and # n (%).

[1] CSF RBC count was significantly higher on day 15 after SAH onset in shunt-dependent patients.

[2] CSF TP was significantly higher on days 5, 11, and 14 after SAH onset in shunt-dependent patients.

[3] Age of patients with EVD-associated ventriculitis is provided.

[4] Age of patients within the non-ventriculitis groups is provided.

[5] Good outcome was defined as a mRS score ≤3, worse outcome as a mRS score >3 determined 3 months after disease onset.

[6] DCI was considered to be present if there was a new CT hypodensity or T2-weighted MRI hyperintensity that was not present on admission imaging or imaging performed within 48 hours of aneurysm occlusion, the lesion had not been adjudicated as being due to surgical or endovascular intervention, and the lesion was not due to EVD placement or intraparenchymal hematoma.

[7] Starting with the first CSF sample collection within 48 hours of SAH onset and extending through 7 days after onset.

Abbreviations: CSF, cerebrospinal fluid; DCI, delayed cerebral ischemia; EVD, external ventricular drainage; ELD, external lumbar drain; FU, follow-up; mRS, modified Rankin Scale; NA, not available; P, prospective; R, retrospective; RBC, CSF red blood cell count; s-SAH, spontaneous subarachnoid hemorrhage; SC, single center; TP, CSF total protein; VPS, ventriculoperitoneal shunt; VS, vasospasm; WBC, CSF white blood cell count
